# Changing Patterns of Microhabitat Utilization by the Threespot Damselfish, *Stegastes planifrons*, on Caribbean Reefs

**DOI:** 10.1371/journal.pone.0010835

**Published:** 2010-05-26

**Authors:** William F. Precht, Richard B. Aronson, Ryan M. Moody, Les Kaufman

**Affiliations:** 1 National Oceanic and Atmospheric Administration (NOAA), Florida Keys National Marine Sanctuary, Key Largo, Florida, United States of America; 2 Department of Biological Sciences, Florida Institute of Technology, Melbourne, Florida, United States of America; 3 Dauphin Island Sea Lab, Dauphin Island, Alabama, United States of America; 4 Boston University Marine Program, Department of Biology, Boston University, Boston, Massachusetts, United States of America; University of Canterbury, New Zealand

## Abstract

**Background:**

The threespot damselfish, *Stegastes planifrons* (Cuvier), is important in mediating interactions among corals, algae, and herbivores on Caribbean coral reefs. The preferred microhabitat of *S. planifrons* is thickets of the branching staghorn coral *Acropora cervicornis.* Within the past few decades, mass mortality of *A. cervicornis* from white-band disease and other factors has rendered this coral a minor ecological component throughout most of its range.

**Methodology/Principal Findings:**

Survey data from Jamaica (heavily fished), Florida and the Bahamas (moderately fished), the Cayman Islands (lightly to moderately fished), and Belize (lightly fished) indicate that distributional patterns of *S. planifrons* are positively correlated with live coral cover and topographic complexity. Our results suggest that species-specific microhabitat preferences and the availability of topographically complex microhabitats are more important than the abundance of predatory fish as proximal controls on *S. planifrons* distribution and abundance.

**Conclusions/Significance:**

The loss of the primary microhabitat of *S. planifrons*—*A. cervicornis*—has forced a shift in the distribution and recruitment of these damselfish onto remaining high-structured corals, especially the *Montastraea annularis* species complex, affecting coral mortality and algal dynamics throughout the Caribbean.

## Introduction

Caribbean coral reefs have changed dramatically over the past few decades [1]. Until the late 1970s, Caribbean reefs displayed a generalized zonation dominated by three common taxa of scleractinian corals, which were the primary builders of reef framework: the branching elkorn coral *Acropora palmata*, the branching staghorn coral *A. cervicornis*, and the massive corals of the *Montastraea annularis* species complex [2], [3]. Since that time coral cover has declined [4], and the pattern of zonation has essentially vanished [5]. The most conspicuous change has been the near-elimination of acroporid corals across the entire region [6]. Several factors have been responsible for the mass mortality of *Acropora*, with white-band disease, predation, and hurricanes ranking as the most significant [1], [6]. Corals of the *M. annularis* complex have also declined on some reefs [7], [8], but the causes have been different than for the *Acropora* species [9]. In this paper, we examine the effects of these shifts in coral assemblage structure on microhabitat utilization by the ecologically significant threespot damselfish, *Stegastes planifrons* (Cuvier). This species of herbivorous fish is important in reef communities of the Caribbean, because it mediates interactions among corals, algae, and other herbivores [10].

Although *S. planifrons* are capable of occupying a number of microhabitats [10]–[14], they prefer thickets of *A. cervicornis* [10], [15], [16]. Before 1980, *S. planifrons* were common residents of shallow and intermediate depths (<30 m) on fore-reef terraces throughout the Caribbean [10]. Because suitable microhabitat was abundant on most Caribbean reefs at the time, it was thought that *S. planifrons* were not at carrying capacity and were, therefore, recruitment-limited [11]. This view may be changing, as live coral cover, especially that of the *Acropora* species, has plummeted in recent decades, greatly reducing the overall habitat available for *S. planifrons*.

Clarke [17] noted that in the Bahamas *S. planifrons* were 20 times more abundant in structurally complex coral thickets, especially thickets of *A. cervicornis*, than in any other microhabitat type. He suggested that *S. planifrons* utilized structurally complex microhabitats generated by *A. cervicornis* to avoid predation. Williams [15] indicated that predation on *S. planifrons* occupying *A. cervicornis* patches was very low. In the absence of *A. cervicornis* threespots exhibited a preference for structurally complex massive corals [12], [18], which presumably also provided some refuge from predators. These secondary, suboptimal microhabitats offered fewer crevices and hiding areas than *A. cervicornis*, and as a result resident damselfish suffered higher mortality [16], [19]. Once territories were established on massive corals, however, *S. planifrons* showed strong site fidelity and long-term survivorship [20]–[23].

**Figure 1:**
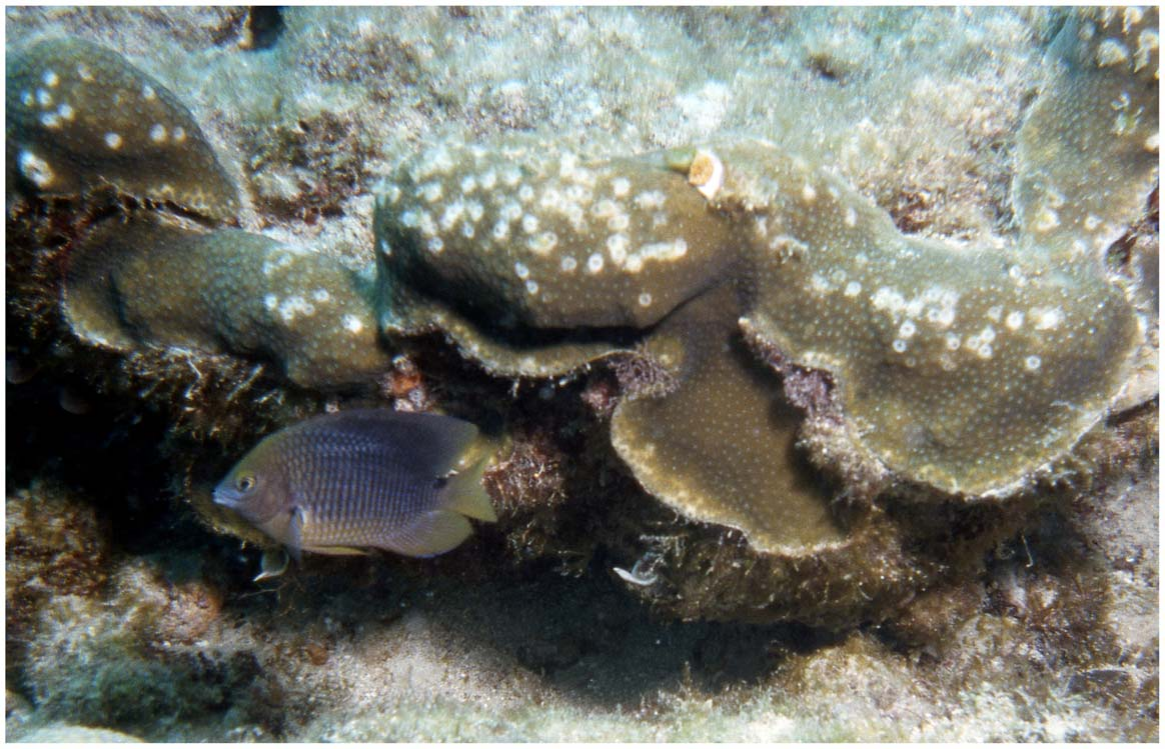
A threespot damselfish, *Stegastes planifrons*, and its territory on a colony of *Monastraea faveolata*. Note the bite-induced lesions of living coral tissue along the margin of the territory. From Carysfort Reef, Florida Keys National Marine Sanctuary; summer 2003; 10 m depth. Photo by WFP.

*S. planifrons* are highly territorial and actively kill scleractinian corals by biting the living tissue and cultivating dense algal lawns on the coral skeletons [10]. Many reef fishes tend algal gardens, but *S. planifrons* are the only Caribbean damselfish so strongly tied to stands of living coral (Figure 1), aggressively defending and guarding their territories against other herbivores. In this respect they represent the extreme expression of a behavior manifested in a host of reef-dwelling pomacentrid species in the genera *Stegastes*, *Dischistodus*, *Hemiglyphidodon*, *Plectroglyphidodon*, *Pomacentrus*, *Microspathodon,* and *Chrysiptera* [24]. Although *S. planifrons* do not appear to kill corals for direct food value [19], the fish clearly benefit from causing coral mortality [10], [25].

### Historical Observations

In the 1970s, prior to the acroporid die-off, Kaufman [10] noted that up to 40% of the surface of the fore-reef terrace at Discovery Bay, Jamaica was covered by algal gardens of *S. planifrons*. At any given time more than 20% of the reef surface contained living coral under attack by *S. planifrons* [26]. Because the life-history strategy of *M. annularis* complex includes slower growth and a more massive skeletal structure than *Acropora* spp., the effects of *S. planifrons* gardens on knobs and pillars of living *M. annularis* complex are more devastating (Figure 1); Kaufman [10] suggested that gardening by *S. planifrons* could actually increase the spatial coverage of healthy stands of *A. cervicornis* by dampening competition from massive corals and by promoting branch fragmentation. Thresher [27] likewise suggested that the activity of *S. planifrons* permitted *A. cervicornis* to dominate, because the branching corals recovered rapidly from their injuries while more massive corals did not. Wellington [28] noted a similar relationship in the eastern Pacific, where the activity of the damselfish *S. acapulcoensis* facilitated the establishment of monospecific stands of branching pocilloporid corals at the expense of massive pavonid colonies.

In 1980, Hurricane Allen struck the north coast of Jamaica, drastically altering reef community structure by breaking and killing most of the branching corals [29]. *S. planifrons*, their territories, and the supporting thickets of *A. cervicornis* were almost entirely eliminated to a depth of ∼12 m on the fore reef at Discovery Bay [26]. After the storm, the density of *S. planifrons* increased in deeper water as the fish relocated and recruited to areas that were still relatively coral-rich. Immigration of mature *S. planifrons* into deeper water was reflected in patterns of coral mortality and microhabitat utilization [26]. *S. planifrons* that recruited to deeper water after the storm generally did not return to shallow water, a direct consequence of the disappearance of physical structure caused by the loss of the branching acroporids. New *S. planifrons* territories established on surviving *A. cervicornis* colonies in shallow water caused significant secondary mortality of the corals [30]. Knowlton et al. [30] noted that when *A. cervicornis* was abundant, predators such as *S. planifrons* generally did not have a detrimental effect; however, when *A. cervicornis* was rare, predation effects could devastate the surviving colonies. Roberts [31] suggested that the continuing negative effect of *S. planifrons* on remnant colonies of *A. cervicornis* may be keeping the coral rare, threatening the long-term prospects for its persistence regionally.

More than 90% of large, robust colonies of the *M. annularis* complex survived Hurricane Allen on the fore reef at Discovery Bay [32]. Columnar growths of *Montastraea* protruded upward from fields of broken and flattened *A. cervicornis* branches, and many surviving *Montastraea* colonies were subsequently colonized by the *S. planifrons* that had lost their territories in *A. cervicornis* thickets (WFP, LK personal observations). This switch caused significant collateral mortality in the remaining population of *M. annularis* complex, as well as subsequent algal overgrowth of the dead colonies [1]. Liddell et al. [33] were the first to suggest that the shift in microhabitat use by *S. planifrons* following the loss of *A. cervicornis* might represent an important new source of mortality for *M. annularis* complex and ultimately affect the production of reef framework.

Were the densities of *S. planifrons* artificially inflated by decades of overfishing, which released the damselfish from predation? To answer this question, Kaufman [34], [35] sampled fossil *A. cervicornis* branches from the Pleistocene Falmouth Formation (∼125 ka) at Rio Bueno, Jamaica. He observed abundant skeletal galls on fossil branches of *A. cervicornis* that had resulted from the bites of *S. planifrons*, suggesting that high densities of threespot territories have long been an attribute of Caribbean reef ecology. The discovery of a living amphipod species found only in *S. planifrons* algal gardens on *A. cervicornis* [36] is further circumstantial evidence that the relationship between *S. planifrons* and *A. cervicornis* is historically rooted and not a recent artifact of Caribbean reef ecology.

### Recent Observations

The recent paucity of *A. cervicornis* throughout the Caribbean has apparently caused a shift in *S. planifrons* from its preferred microhabitat to secondary microhabitats. *S. planifrons* are familiar occupants of any microhabitat that is structurally complex with abundant vertical fissures. These include columnar morphologies of the *M. annularis* complex [14], [37], [38]. In Florida, Eakin [39] observed that, in the absence of live branching corals, juvenile *S. planifrons* preferentially recruited to living *Montastraea* colonies. These observations strongly suggest that *M. annularis* complex has now become the primary microhabitat of *S. planifrons* on fore-reef terraces throughout the Caribbean.

Reef-fish assemblages have changed concomitantly with coral assemblages. Predators, especially groupers (Serranidae) and snappers (Lutjanidae), have declined in recent decades due to overfishing and habitat loss [40]–[42]. A number of authors have asserted a causal chain leading from overfishing, to reduced densities of predators, to enhanced damselfish densities, to increased coral mortality, and thence to increased algal cover. Vicente [43] and Hernandez-Delgado [37], for example, attributed algal overgrowth of corals in Puerto Rico to overfishing, which in their view released *S. planifrons* from predation and allowed them to kill the corals. Ogden [44], citing results from the Caribbean Coastal Marine Productivity (CARICOMP) Program, implicated overfishing as causing enhanced abundance of damselfishes throughout the region. He suggested that increases in damselfish densities have overwhelmed the capacity of the corals to counteract their impacts, resulting in the decline of coral populations and the smothering of reefscapes with vast algal lawns. Others have voiced similar opinions in the scientific literature and the popular news media [45]–[47].

A major factor confounding the overfishing hypothesis is the loss of reef fish, including the predators of damselfishes and the damselfish themselves, caused by mass coral mortality and the consequent loss of reef structure [48]–[50]. Although it stands to reason that fewer predators could result in higher numbers of algal-gardening damselfish [51] or alterations in territorial dynamics [52], these expectations are predicated on the assumption that *S. planifrons* were/are in fact predator-limited. An alternative hypothesis is that *S. planifrons* populations are limited ultimately by predators but proximally by the availability of microhabitat: they have evolved to evade predation by remaining closely associated with appropriate structural refugia. If this alternative hypothesis is correct, reducing predator abundance could result in increased survival of non-territorial or peripheral individuals, but the density of coral-killing algal gardeners should remain approximately the same over a broad range of predation intensities. There has never been a formal test of the hypothesis that the density of territorial *S. planifrons* is predator-limited when sufficient preferred habitat is available.

### Hypotheses

The null hypothesis is that there is no proximal effect of piscivorous fishes on the abundance of *S. planifrons*. If on the other hand predation by piscivorous fishes controls the abundance and distribution of *S. planifrons*, reefs with higher fishing pressure should have higher densities of *S. planifrons* than reefs with lower fishing pressure. Under this scenario *S. planifrons* territories should have spread into all available microhabitats on overfished reefs, causing coral mortality and the massive proliferation of algal gardens. If microhabitat availability controls the abundance of *S. planifrons*, the loss of *A. cervicornis* should have caused a shift in microhabitat use without necessarily increasing the overall densities of these damselfish on overfished reefs. The shift to secondary, suboptimal microhabitats that were previously devoid of *S. planifrons* should also have resulted in coral mortality and proliferation of algal gardens. A third alternative is the combined action of the two processes: both predatory release and microhabitat availability controlling the distribution and abundance of *S. planifrons*.

## Methods

### Study Areas

During the period 1998–2001, we compared sites in Jamaica (heavily fished), Florida and the Bahamas (moderately fished), the Cayman Islands (lightly to moderately fished), and Belize (lightly fished) to test the alternative hypotheses (Table 1). We selected study sites based on the following criteria: (a) sites were chosen along a gradient of fishing pressure; (b) all sites were located in fore-reef habitats at 10–15 m depth; (c) all sites were known to have had abundant stands of *A. cervicornis* in the recent past; and (d) *A. cervicornis* was either rare or absent at each site during the study period. Differences in fishing pressure were ascertained from the published literature [40], [41], [53]–[57], as well as from interviews with fisherman, dive operators, reef scientists, and site managers from these locations.

**Table 1:** Descriptive information for the ten sites used in the study.

Site Designation	Coordinates	Sampling Year	Depth (m)
Grand Cayman North	19° 23.46′ N, 81° 23.03′ W	2001	12
Grand Cayman South	19° 15.21′ N, 81° 23.03′ W	2001	12
Goulding Cay, Bahamas	25° 01.15′ N. 77° 34.04′ W	1998	12
LTS Reef, Discovery Bay, Jamaica	18° 28.21′ N, 77° 24.47′ W	1998	10
Pear Tree Bottom, Jamaica	18° 27.80′ N, 77° 21.69′ W	1998	10
French Reef, Florida Keys	25° 02.06′ N, 80° 21.00′ W	2000	10
Carysfort Reef, Florida Keys	25° 13.80′ N, 80° 12.74′ W	2000	10
Key Largo Dry Rocks, Florida Keys	25° 07.45′ N, 80° 17.80′ W	2000	10
Tobacco Reef, Belize	16° 52.48′ N, 88° 03.47′ W	2001	12
Carrie Bow Cay, Belize	16° 48.21′ N, 88° 04.42′ W	2001	15

One to three study sites were established at each survey location. At each site, six 25-m surveyor's tapes were laid haphazardly. A diver swam along each transect, identifying and counting fishes within 1 m on either side of the tape. Fish species were categorized as: (a) *S. planifrons*; (b) pomacentrid species other than *S. planifrons*; (c) herbivores other than damselfish; or (d) piscivores. The diver then swam back over the transect line, recording the sessile organism or substratum type beneath each 10-cm mark on the tape. Finally, the diver swam the transect a third time, recording all regular echinoids within 1 m on either side.

Structural complexity, or topographic heterogeneity, was measured by conforming a 5-m length of brass chain (links 17 mm long) to the substratum along the central portion of each 25-m tape, beginning 10 m from the start of the transect. An index of structural complexity was calculated as *C* = 1−*d/l*, where *d* is the horizontal distance covered by the chain when conformed to the substratum and *l* is the length of the chain when fully extended [58].

### Statistical Analysis

We used principal components analysis (PCA) on mean values from the six 25-m transects at each study site to determine the proportion of among-site variance in the abundance of *S. planifrons* attributable to: (a) the cover of *M. annularis* complex (which consisted exclusively of *M. annularis* sensu stricto and *M. faveolata*); (b) the cover of *A. cervicornis*; (c) the cover of living hard corals other than *M. annularis* complex and *A. cervicornis* (‘other hard corals’); (d) the structural complexity of the benthos; (e) the water depth; (f) the abundance of piscivorous fish; (g) the abundance of non-pomacentrid herbivorous fish (‘other herbivorous fishes’); and (h) the abundance of pomacentrids other than *S. planifrons* (‘other damselfishes’). Regular echinoids were exceedingly rare in the transects and were not included in the PCA. Significant eigenvector loadings were identified by performing Pearson correlation analyses between the original independent variables and their corresponding eigenvectors for each PC [59].

PCA requires that all samples for each independent variable be drawn from a normal distribution. To meet this requirement, point-counts of *A. cervicornis* were transformed using the function {*Y* = −1/*x*}. The remaining seven variables did not require data transformation.

We also performed separate linear-regression analyses of *S. planifrons* abundance versus: (a) the abundance of piscivores; (b) the cover of *M. annularis* complex; (c) the structural complexity of the substratum; and (d) the total cover of living hard corals (including *M. annularis* complex and *A. cervicornis*). Total hard-coral cover was calculated as the sum of point-counts for *M. annularis* complex, *A. cervicornis*, and all other species of hard corals. The regressions were run to determine whether the variables of primary interest had significant predictive power in explaining the abundance of *S. planifrons* among sites. *S. planifrons* abundance and all independent variables were log_10_-transformed prior to regression analysis.

## Results

**Figure 2:**
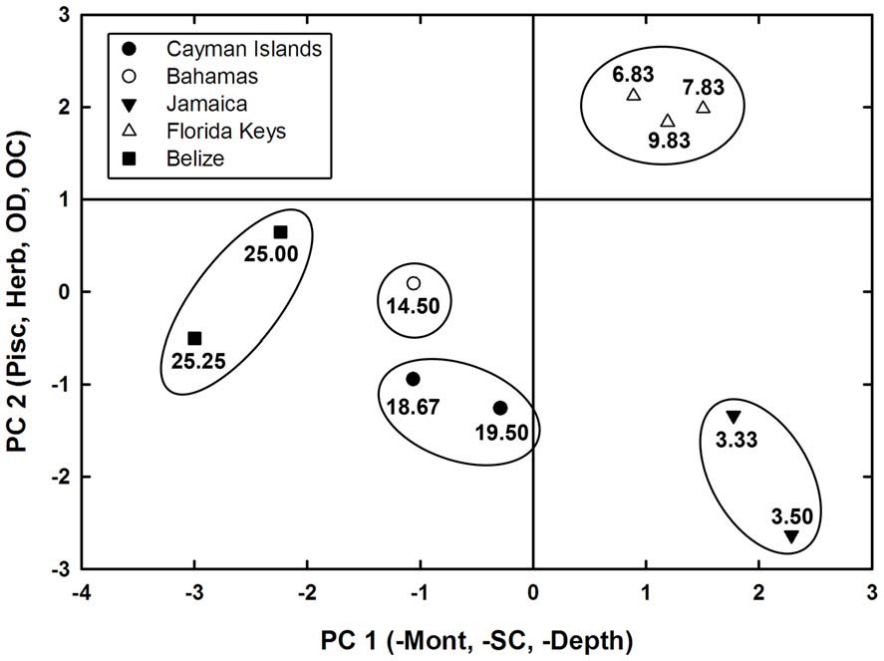
Scatterplot of site-scores on the first and second principal components. These two axes represent 73.4% of the total variation in the correlation matrix. Independent variables listed on each axis indicate variables with significant eigenvector loadings. Abbreviations for variables are listed in Table 2. Mean *S. planifrons* densities are given for each site (*n* = 6 transects per site).

Three principal components (PCs) extracted from the correlation matrix explained 87.3% of the variability among sites (Figure 2, Table 2). PC1 and PC2 explained 73.4% of the variability, and PC4 contributed 13.9%. The remaining five PCs explained 12.7% of the total variance and were not considered further.

**Table 2:** Eigenvectors and eigenvalues for the principal component analysis of eight independent variables collected at the 12 sites.

Original Variables	PC1	PC2	PC3
Piscivores (Pisc)	0.230	0.480	−0.392
Other Herbivorous Fishes (Herb)	−0.346	0.397	−0.143
Other Damselfish (OD)	0.055	0.545	−0.268
*Montastraea annularis* (Mont)	−0.539	0.057	−0.104
*Acropora cervicornis* (Acrop)	0.057	−0.282	−0.755
Other Hard Corals (OC)	−0.055	−0.443	0.404
Structural Complexity (SC)	−0.519	0.125	−0.092
Depth (Depth)	−0.509	−0.141	−0.034
Eigenvalue (%)	38.9	32.9	15.8

PC1 accounted for 38.9% of the variability in the data set, yielding significantly negative eigenvector loadings for point-counts of *M. annularis* complex, structural complexity, and depth (Table 2). Sites with lower PC1 scores had higher point-counts of *M. annularis* complex, higher structural complexities, and slightly deeper depths. *M. annularis* complex was the most abundant coral taxon at each site, with an overall mean proportional contribution to total living-coral point-counts of 0.53±0.04 SE (range 0.32–0.78).

PC2 accounted for 32.9% of the total variance in the data set and yielded significantly positive eigenvector loadings for mean counts of piscivores; non-pomacentrid, other herbivores; other damselfishes; and point-counts of other corals. Positive eigenvector loadings were generated for all functional groups of fish, indicating that piscivores did not have a negative impact on either other pomacentrids or other herbivorous fishes. Sites with higher PC2 scores exhibited higher abundances of piscivores, other herbivorous fishes, other damselfishes, and other hard corals. PC3 explained 15.8% of the variance in the data set and represented the abundance of *A. cervicornis* at each location.

The piscivores detected in the transects at all sites were small to intermediate in size, at 20–50 cm standard length. Belt transects are poor estimators of the abundance of large, wide-ranging, predatory fishes, as compared to smaller, site-attached fishes [60], [61]. We noted the presence/absence of larger piscivores, including sharks, barracuda, groupers, snappers, and jacks, adjacent to our transects; at all study sites these fishes were uncommon to rare and, therefore, assumed not to be of primary importance to our analysis.We focused on small- to intermediate-sized piscivores, which included the smaller serranids and lutjanids, because they are the primary predators of adult and juvenile *S. planifrons* [47], [62]. These smaller predators could themselves have been released by the overfishing of larger predators; thus, fishing pressure could actually be expected to result in fewer, rather than more, damselfishes. For example, Stallings [63] found that the harvesting and depletion of Nassau grouper, *Epinephelus striatus*, allowed two smaller-bodied, intermediate predators (coney and graysby groupers, *Cephalopholis fulva* and *C. cruentata*) to proliferate, which in turn had strong negative effects on their prey. Our data, however, do not support such a hypothesis.

Sites with low PC1 loadings—high point-counts of *M. annularis* complex, high levels of structural complexity, and deeper depths—exhibited high densities of *S. planifrons*; these sites were located in Belize, the Bahamas, and the Cayman Islands. Sites with high PC1 loadings exhibited low and intermediate densities of *S. planifrons*; these sites were located in Jamaica and the Florida Keys (Figure 2). Sites exhibiting intermediate densities of *S. planifrons*, all of which were located in Florida, exhibited PC1 loadings >1.

Positive PC2 loadings, denoting high abundances of piscivores, other herbivorous fishes, other damselfishes, other corals, were associated with intermediate to high abundances of *S. planifrons*. We interpret this to mean that the site groupings reflect differences in overall biotic composition among locations rather than the impact of one particular PC2 variable on the abundance of *S. planifrons*. Thus, counts of *S. planifrons* did not decline monotonically as abundances of piscivores and other damselfishes increased but instead tracked the availability of coral-generated habitat complexity.

**Figure 3:**
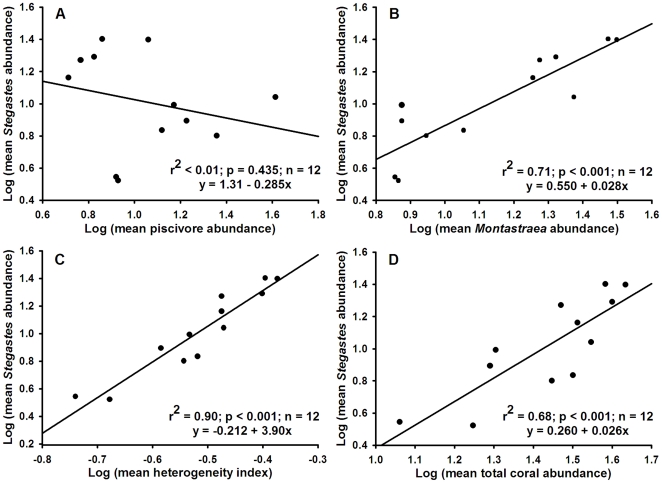
Relationships between key parameters and the abundance of *S. planifrons*. The abundance of *S. planifrons* regressed on: (A) piscivore abundance; (B) proportional cover of the *Montastraea annularis* species complex; (C) structural complexity; and (D) proportional cover of total hard corals. The coordinates of each point are the log-transformed means of the transects within a site.

In agreement with our interpretation of PC1, regression analysis did not reveal a significant relationship between the abundance of *S. planifrons* and piscivores among our study sites (*r* ^2^<0.01; *p* = 0.435; *n* = 12; Figure 3A). We did, however, detect a strong relationship between the abundances of *S. planifrons* and *M. annularis* complex (*r* ^2^ = 0.71; *p*<0.001; *n* = 12; Figure 3B), structural complexity (*r* ^2^ = 0.90; *p*<0.001; *n* = 12; Figure 3C), and total coral counts (all hard corals, including *M. annularis* complex and *A. cervicornis*: *r* ^2^ = 0.68; *p*<0.001; *n* = 12; Figure 3D). These findings are consistent with the high PC1 loadings for each of these variables and consistent with the fact that *M. annularis* complex is both a dominant member of the coral assemblage at each site and currently the preferred microhabitat of *S. planifrons* at these depths.

## Discussion

Our data strongly suggest that the availability of appropriate microhabitat is the primary determinant of the population density of *S. planifrons*. The negative correlation between the abundance of (small- to intermediate-sized) piscivores and the abundance of *S. planifrons*, expected under the hypothesis of predatory control, was negligible and non-significant. The evolution of microhabitat preference by *S. planifrons* is likely to have been at least in part a consequence of predation pressure [64]; however, the survey data do not support the hypothesis that current densities of piscivorous fishes determine current densities of *S. planifrons*, a conclusion borne out by other studies in Curaçao and the Florida Keys [65].

In a long-term monitoring program in the U.S. Virgin Islands, smaller serranids and lutjanids increased over an 18-year interval, while larger piscivores remained rare [66]. Population densities of *S. planifrons* increased during the same period. The increase in small- to intermediate-sized predators, however, should have resulted in fewer damselfish. Clearly the abundances of piscivores and *S. planifrons* were decoupled. Likewise, whether or not fishing pressure enhanced the abundances of small- to intermediate-sized piscivores at any of our sites is moot from the standpoint of the abundance of threespot damselfish.

Williams [67] performed exclusion experiments in Jamaica to test the effect of predation on adult *S. planifrons*. She found no significant change in numbers of *S. planifrons* in the absence of predation. Likewise, She noted that when *A. cervicornis* was abundant predators did not have a strongly detrimental effect on *S. planifrons* populations [15], [67]. On the Great Barrier Reef, artificial reefs constructed from the high-complexity coral *Pocillopora damicornis* supported the same numbers of juvenile damselfish when predators were abundant as when predators were absent [68].

The highest densities of piscivores and damselfishes other than *S. planifrons* were found in the Florida Keys and could have been related to the local protection afforded no-take reserves. *S. planifrons* occurred at intermediate abundances in Florida, despite the negative effects of predation and competition that might have been expected (*contra* 69).

Prior to the demise of the acroporids, Bohnsack [70] tested the predation hypothesis in the Florida Keys. In contrast to our results, he found that reefs with high fishing pressure *did* have significantly higher numbers of *S. planifrons* than reference reefs with lower fishing pressure and higher numbers of piscivores. The results, however, were confounded by differences in habitat type between his study reefs: the protected sites with higher numbers of *S. planifrons* were dominated by *A. cervicornis*, whereas the sites with lower numbers of *S. planifrons* were dominated by an assemblage of head corals. Bohnsack [70] recognized this problem and was careful to note, “Stating that piscivorous predation is an important factor controlling community structure of reef fishes based on present evidence would be premature.”

Perhaps the most persuasive evidence against monolithic predator-limitation of *S. planifrons* is that even on the north and west coasts of Jamaica, an extreme example of a chronically overfished situation [71], *S. planifrons* exhibited high microhabitat specificity. Population and territory spillage onto hemispheric or horizontal-foliose corals occurred only where these non-preferred microhabitats were immediately adjacent to patches of preferred microhabitat [10], [72]. When acroporids virtually disappeared, threespots moved onto very specific secondary and tertiaty microhabitats (*M. annularis* complex and *Porites porites*; [26]). Where only low-relief fields of coral rubble remained, the density of adult *S. planifrons* was drastically reduced [see also 73]. The greatly expanded small-coral and coral-rubble microhabitats were heavily colonized by two other damslefish species that became more prevalent: *Stegastes diencaeus*, which is a less active gardener than *S. planifrons*, and *S. partitus*, which is a planktivore and not an algal gardener (LK personal observation). Gladfelter et al. [74] also noted a decrease in numbers of *S. planifrons* in St. Croix after Hurricane Hugo as a direct consequence of the physical loss of microhabitat.

On a regional level, the loss of structural complexity caused by the Caribbean-wide mass mortality of *A. cervicornis* in the late 1970s and 1980s [50] reduced the total amount of shelter available to *S. planifrons*. The damselfish relocated or recruited to remaining high-structured living corals, especially *M. annularis* complex. *S. planifrons* predation on the living coral tissue of these secondary microhabitats has been chronic and intense, resulting in extensive coral mortality and proliferation of algal gardens (Figure 1).

Wellington [75] demonstrated that loss of structurally complex branching corals from disturbance resulted in the relocation of *S. acapulcoensis* to secondary microhabitats, with lethal consequences for massive corals. Monospecific stands of *Pocillopora damicornis*, the microhabitat preferred by the damselfish [28], were killed in the Gulf of Panama by the 1982–1983 El Niño event. As structural complexity was reduced by bioerosion in the years following this disturbance, *S. acapulcoensis* colonized the massive coral *Gardineroseris planulata*. Colonies of *Gardineroseris* that had been monitored for 14 years were free of *S. acapulcoensis* before the El Niño event. These colonies subsequently suffered substantial mortality from *S. acapulcoensis* [7], [76].

These examples show that when the preferred microhabitats of territorial damselfish are abundant, there is little collateral mortality or algal overgrowth on secondary microhabitats. When the preferred microhabitats are eliminated by mortality of the engineer species, which is to say branching corals, the impact on secondary microhabitats can be dramatic and intense. Because massive corals grow more slowly than branching corals, episodes of mass mortality of branching corals inhabited by algal gardeners may leave an enduring imprint on community structure, continuing long after the branching corals reestablish themselves and the damselfishes move back into them and away from their suboptimal microhabitats.

Observations from Pleistocene coral assemblages confirm that *S. planifrons* had been abundant and *A. cervicornis* had been their preferred microhabitat in the tropical Atlantic for a long time prior to any human interference [34], [35]. Other paleoecological evidence indicates the recent mass mortality of Caribbean acroporids to be a novel event in the late Holocene [77]. Although some populations of *Acropora* spp. have been extirpated locally, the two species are surviving regionally and may yet recover to their former large population sizes [78]. Caribbean-wide regeneration and recovery of the *S. planifrons*–*Acropora* relationship could take decades or centuries. Locally, however, acroporid restoration could yield improved survivorship for massive corals by allowing the *S. planifrons* to relocate to their preferred microhabitat; this idea is currently being tested by two of us (LSK and WFP) in the Florida Keys. In the meantime, further community disintegration should be expected as *S. planifrons* continue their turf wars on slow-growing, long-lived, massive corals.
